# Dexmedetomidine attenuates acute stress-impaired learning and memory in mice by maintaining the homeostasis of intestinal flora

**DOI:** 10.1186/s40001-024-01832-5

**Published:** 2024-05-06

**Authors:** Hao Feng, Xing Hu, Yizi Lin, Jingni Xiao, Chao Dai, Zhaolan Hu, Hao Feng, Jiao Qin, Li Chen

**Affiliations:** 1 Department of Dermatology, Hunan Provincial People’s Hospital (The First Affiliated Hospital of Hunan Normal University), Changsha, Hunan 410000 People’s Republic of China; 2 Department of Radiology, The Third Affiliated Hospital of Wenzhou Medical University, Wenzhou, Zhejiang 325200 People’s Republic of China; 3 Department of Nephrology, Hengyang Medical School, University of South China Affiliated Changsha Central Hospital, No. 161 Shaoshan South Road, Changsha, Hunan 410004 People’s Republic of China; 4 Department of Anesthesiology, The Second Xiangya Hospital, Central South University, 139 Ren-Min Central Road, Changsha City, Hunan 410011 People’s Republic of China; 5 State Key Laboratory of Developmental Biology of Freshwater Fish, College of Life Science, Hunan Normal University, Changsha, 410081 People’s Republic of China; 6 Department of Anesthesiology, The Third Affiliated Hospital of Wenzhou Medical University, No.108 Wansong Road, Wenzhou, Zhejiang 325200 People’s Republic of China

**Keywords:** Dexmedetomidine, Intestinal flora, Learning and memory, Acute stress

## Abstract

Dexmedetomidine (Dex) has been used in surgery to improve patients' postoperative cognitive function. However, the role of Dex in stress-induced anxiety-like behaviors and cognitive impairment is still unclear. In this study, we tested the role of Dex in anxiety-like behavior and cognitive impairment induced by acute restrictive stress and analyzed the alterations of the intestinal flora to explore the possible mechanism. Behavioral and cognitive tests, including open field test, elevated plus-maze test, novel object recognition test, and Barnes maze test, were performed. Intestinal gut Microbe 16S rRNA sequencing was analyzed. We found that intraperitoneal injection of Dex significantly improved acute restrictive stress-induced anxiety-like behavior, recognition, and memory impairment. After habituation in the environment, mice (male, 8 weeks, 18–23 g) were randomly divided into a control group (control, *N* = 10), dexmedetomidine group (Dex, *N* = 10), AS with normal saline group (AS + NS, *N* = 10) and AS with dexmedetomidine group (AS + Dex, *N* = 10). By the analysis of intestinal flora, we found that acute stress caused intestinal flora disorder in mice. Dex intervention changed the composition of the intestinal flora of acute stress mice, stabilized the ecology of the intestinal flora, and significantly increased the levels of *Blautia* (A genus of anaerobic bacteria) and *Coprobacillus*. These findings suggest that Dex attenuates acute stress-impaired learning and memory in mice by maintaining the homeostasis of intestinal flora.

## Introduction

Dexmedetomidine (Dex) has been used in surgery to improve patients' postoperative cognitive function, such as short-term memory and recognition ability impairment [1]. Dex improves cognitive function by protecting the injury of the nervous system and immune system from inflammation [2, 3]. Dex treatment can inhibit the expression of inflammatory factors such as interleukin 6, and tumor necrosis factor α to reduce the nervous system damage caused by intraoperative cerebral ischemia and hypoxia [4, 5].

However, the role and mechanism of Dex in stress-induced anxiety-like behaviors and cognitive impairment is still unclear. Recent studies have shown that Dex could alleviate sleep-restriction-inhibited splenic CD8 + T cell activity via modulating gut microbiota, which acted through subdiaphragmatic vagus nerve [6], suggesting that Dex may protect the stress response through intestinal flora [7].

The gut and brain are interconnected through neural, endocrine, and immune pathways. Recent studies have shown that gut microbes are involved in the functional response of the gut–brain axis and play an important role in the communication of information between the gut and brain [8, 9]. Stress can activate the activity of the hypothalamus–pituitary–adrenal axis (HPA) further activating the neuroendocrine system. Acute or chronic stress can cause changes in the host's gut microbes and intestinal physiological functions, as well as impairment of cognitive functions [10]. For example, infecting wild-type mice with *Citrobacter rodentium* did not affect their memory and cognition, but applying acute water avoidance stress to the infected mice resulted in a decrease in non-spatial recognition memory and working memory. One week before *Citrobacter rodentium* infection, probiotic intervention effectively prevented stress-induced impairment in cognitive behavior [11]. However, whether Dex improves stress-induced cognitive impairment through intestinal flora is still unclear.

In this study, we aim to explore the role of Dex in anxiety-like behavior and cognitive impairment induced by acute restrictive stress. We found that intraperitoneal injection of Dex significantly improved acute restrictive stress-induced anxiety-like behavior, recognition, and memory impairment. By the analysis of intestinal flora, we found that acute stress caused intestinal flora disorder in mice. Dex intervention improved the flora disorder to a certain extent. These findings suggest that Dex may improve the acute stress-induced anxiety-like behavior and cognitive impairment by maintaining the homeostasis of the intestinal flora.

## Materials and methods

### Animals

C57BL/6 mice (male, 8 weeks, 18–23 g) were purchased from Laboratory Animal Co. Ltd. of Slack King (Longping Sci-tech Park, Changsha, China). Animals were housed with a 4 per cage with a 12 h light/dark cycle at a constant temperature (22–24 °C) and humidity-controlled (50 ± 5%) animal facility, with food and water ad lib. All animal experiments were conducted in compliance with Chinese guidelines for the care and use of laboratory animals and were approved by the Ethics Review Committee for Animal Experimentation of the University of South China (NO. USC202212xs49, Hengyang, China). The mice were euthanized with an overdose anesthetizing of sodium pentobarbitone (90 mg/kg, intraperitoneal injection). All efforts were made to minimize suffering and the number of mice used.

### Acute stress (AS) and dexmedetomidine (Dex) treatment

After habituation in the environment, mice were randomly divided into a control group (control, *N* = 10), dexmedetomidine group (Dex, *N* = 10), AS with normal saline group (AS + NS, *N* = 10), and AS with dexmedetomidine group (AS + Dex, *N* = 10). The control group and Dex group mice were handled twice on the experimental day, while the AS group mice were placed in a 50-ml centrifuge tube for a single session of 2 h [12]. The restrained mice were returned to their home cages. Then, the Dex group and AS + Dex group mice were immediately intraperitoneal (i.p.) injected with 10 μg/kg of DEX (H20090248, Jiangsu Hengrui iPharmaceutical Co., Ltd., China) for 30 min. The AS + NS group mice were treated with the same dose of normal saline.

### Behavioral and cognitive tests

All behavioral procedures were implemented from 9 AM to 4 PM in a sound-isolated room. Tests were performed after Dex or normal saline treatment for 30 min and recorded by the same experimenter blindly.

#### Open field test (OFT)

The open field test (OFT) was performed in a black polyester resin chamber (50 × 50 × 50 cm). The mouse was first sited in the center of the arena and free to explore for 5 min; the total travel distance of motility and the time spent in the central square were recorded and analyzed by Viewpoint Video Tracking Software (Viewpoint Behavior Technology, Lyon, France).

#### Elevated plus-maze test (EPM)

The elevated plus maze is composed of two open arms and two closed arms, which are perpendicular to each other in a cross (arm width 5 cm, arm length 35 cm, closed arm height 15 cm, height above the ground about 50 cm). Before each experiment, the maze was cleaned with alcohol to remove the urine and feces left by the last animal in the maze. The experimental animals were transported to the special temporary cages in the behavioral laboratory in advance to adapt to the environment for about 3 h to reduce animal stress. When the experiment was beginning, the mouse was taken out of the cage, and the experimental animal should face away from the experimenter. The mouse was gently placed in the central area of the maze facing the open arm. The experiment lasted 5 min and used Viewpoint Video Tracking Software (Viewpoint Behavior Technology, Lyon, France) to track the animal’s trajectory movement in the elevated plus maze, which automatically calculated the indicators, including the number of times to enter the open arm and the spent time in the open arm.

#### Novel object recognition test (NOR)

A novel object recognition test (NOR) was carried out in a black polyester resin chamber (50 × 50 × 50 cm). Before the test, mice were habituated in this box for 5 min without any objects. After that, the mouse was placed in the same box and exposed to two identical objects to familiarize the object for 5 min (training session). Then each mouse was returned to their cage after training. Each mouse was allowed to explore both the familiar object and a completely different object (novel object) for 5 min (test session) after 30 min. The time spent exploring familiar and novel objects was recorded and analyzed by Viewpoint Video Tracking Software (Viewpoint Behavior Technology, Lyon, France). A recognition index was defined and calculated as the previous paper [13].

#### Barnes maze test

The spatial learning and memory ability of the mice were performed by the Barnes maze test. The details of the experimental device and the method as previously reported [14]. The mice were first habituated in the experimental room environment for 2 h and trained 3 times per day, within 15 min in two training intervals, for 3 days to guide the learning process. After acquisition training, the probe trial was performed as follows: the mice were placed in the center of the platform in a dark box under a dark environment. After 15 s, the mice were released and guided straight into the target cage for 1 min. Then, the mice were released and allowed to freely explore for 3 min to find the target box, recording the number of mistakes in the search process and the latency time into the target box.

### Intestinal gut microbe 16S rRNA sequencing analysis

The total bacterial genomic DNA was extracted using the OMEGA soil kit following the manufacturer’s instructions. The DNA concentration and purity were determined using a NanoDrop 2000 spectrophotometer (Thermo Fisher Scientific, USA). The quality of the DNA extraction was assessed by electrophoresis using a 1% agarose gel. The V3–V4 variable region of the 16S rRNA gene was used for PCR amplification using the forward primer (5′-ACTCCTACGGGAGGCAGCAG-3′) and the reverse primer (5′-GGACTACHVGGGTWTCTAAT-3′). The resulting PCR amplicons were purified with Agencourt AMPure Beads (Beckman Coulter, Indianapolis, IN) and quantified using the PicoGreen dsDNA Assay Kit (Invitrogen, Carlsbad, CA, USA). After the individual quantification step, amplicons were pooled in equal amounts, and pair-end 2 × 300 bp sequencing was performed using the Illumina MiSeq platform with MiSeq Reagent Kit V3 at Shanghai Personal Biotechnology Co., Ltd (Shanghai, China).

The Quantitative Insights into Microbial Ecology (QIIME, v1.8.0) pipeline was employed to process the sequencing data. Briefly, raw sequencing reads with exact matches to the barcodes were assigned to respective samples and identified as valid sequences. The low-quality sequences were filtered using the following criteria: sequences that had a length of < 150 bp, sequences that had average Phred scores of < 20, sequences that contained ambiguous bases, and sequences that contained mononucleotide repeats of > 8 bp. Paired-end reads were assembled using FLASH.

After chimera detection, the amplicon sequence variant (ASV) with 99% similarity was classified as one operational taxonomic unit (OTU) to obtain the OTU classification information at 97% sequence identity by UCLUST. The OTUs were classified using the RDP classifier to obtain their numbers at different taxonomic levels.

A representative sequence was selected from each OTU using default parameters. OTU taxonomic classification was conducted by BLAST searching the representative sequences set against the Greengenes Database using the best hit. Beta diversity was calculated according to the principal coordinate analysis (PCoA), and orthogonal partial least squares–discriminant analysis (OPLS–DA) was performed to analyze the sample types corresponding to microbial communities [15]. The linear discriminant analysis (LDA) effect size (LEfSe) was further used to identify the dominant bacterial with differences in abundance among control, Dex, AS + NS, and AS + Dex groups using the Kruskal–Wallis, LEfSe and DEseq2 methods and adjusted the *P* value using the Benjamini–Hochberg method [16, 17].

To determine the abundance and homogeneity of the sample species composition, we calculated the alpha diversity indices including the observed OTUs, Shannon index, and Faith's phylogenetic diversity index, and compared the differences in the alpha diversity among groups. The observed OTUs were further generated to record the abundance of each OTU in each sample and the taxonomy of these OTUs. OTUs containing less than 0.001% of total sequences across all samples were discarded. To minimize the difference in sequencing depth across samples, an averaged, rounded rarefied OTU table was generated by averaging 100 evenly re-sampled OTU subsets under 90% of the minimum sequencing depth for further analysis at Shanghai Personal Biotechnology Co., Ltd (Shanghai, China).

### Statistical analysis

All the experiments were repeated independently three times. Data are expressed as mean ± SEM. One-way analysis of variance or two-way analysis of variance was used for statistical analysis. *p* values were accepted as significantly different at *p* < 0.05. The statistical analysis was performed using GraphPad Prism 7.0 (San Diego, United States).

## Results

### Dex protected against AS-induced learning and memory impairment

**Fig.1:**
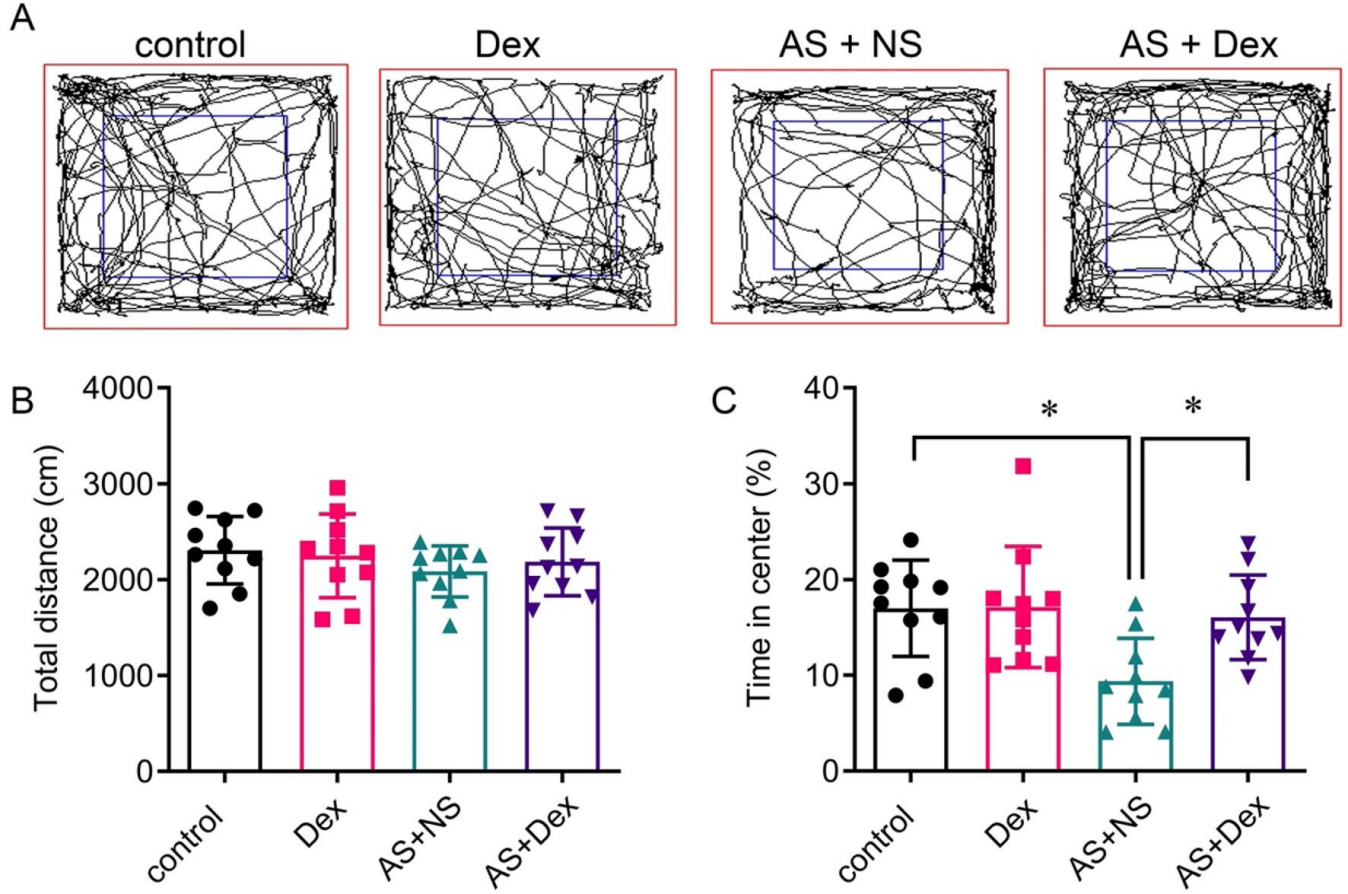
Dex attenuated anxiety behavior of the acute stress mouse. **A** Representative traces in the open field test. **B**, **C** Total distance and the time spent in the center field of the open field test (*n* = 10/group). Data are expressed as mean ± SEM. *p < 0.05

**Fig. 2:**
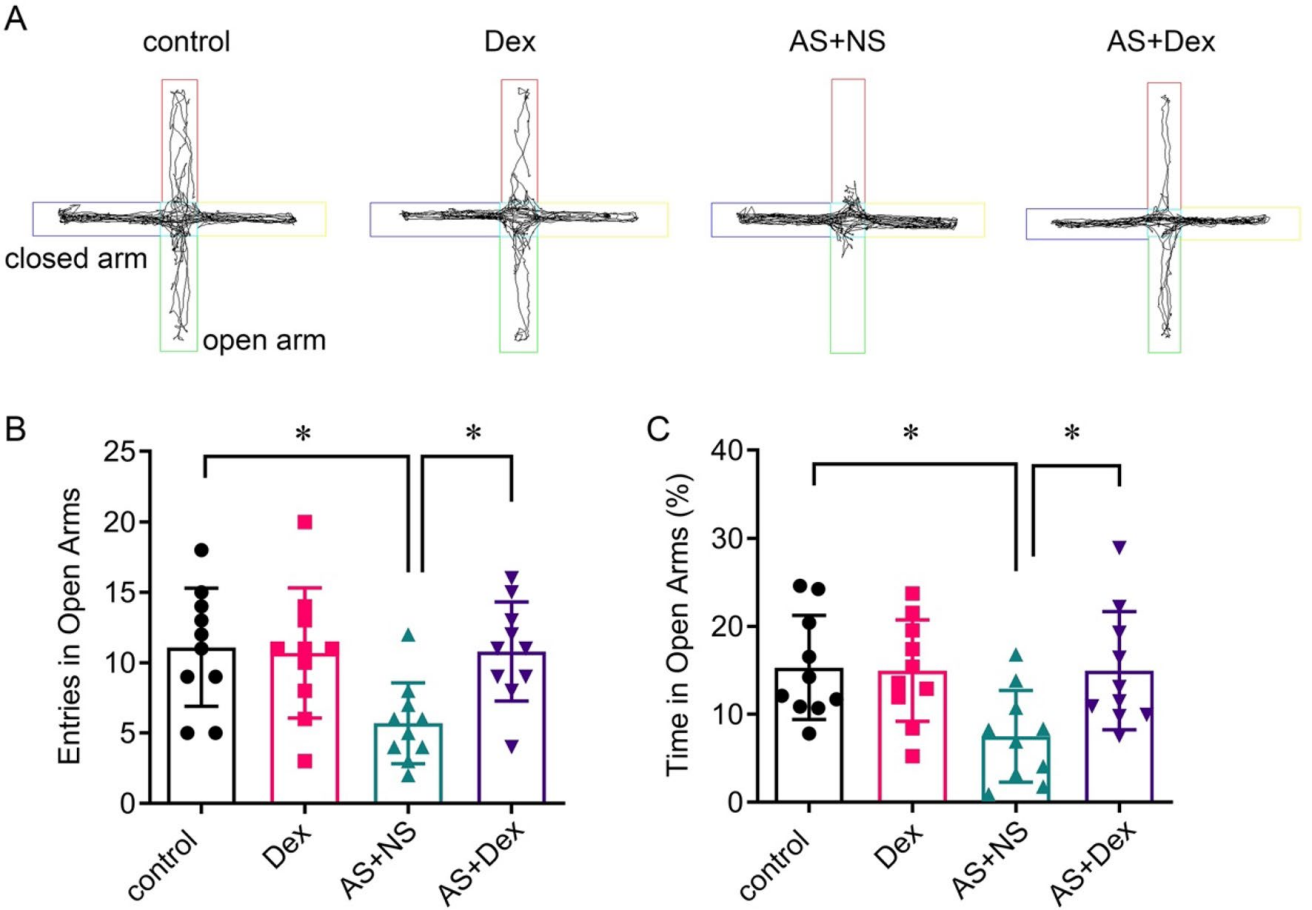
Dex attenuated spatial learning and memory behavior of the acute stress mouse by the Y maze test. **A** Representative traces in the Y maze test. **C** Entries and percentage of time mice stay in the open arm in the Y maze test (n = 10/group). Data are expressed as mean ± SEM. *p < 0.05

The AS + Dex group mice were treated with Dex (10 μg/kg, i.p.) or the equal volume normal saline after AS induced 2 h. The Dex group mice were injected with the same dose of Dex without AS treatment as a parallel experiment. To evaluate the anxiety-like behavior and the learning and memory behavior, OFT, EPM, NOR, and Barnes maze tests were performed in AS-induced mice. The OFT results showed that the total distances traveled had no significant difference in the four groups (*p* > 0.05, Fig. 1A, B). The percentage of time spent in the center square of the AS group was decreased, while Dex treatment significantly increased the time to about 20% (*p* < 0.05, Fig. 1C). In addition, we found that AS decreased the entries and percentage of time in open arms via EPM test, which were attenuated by Dex treatment (Fig. 2A–C).

**Fig. 3:**
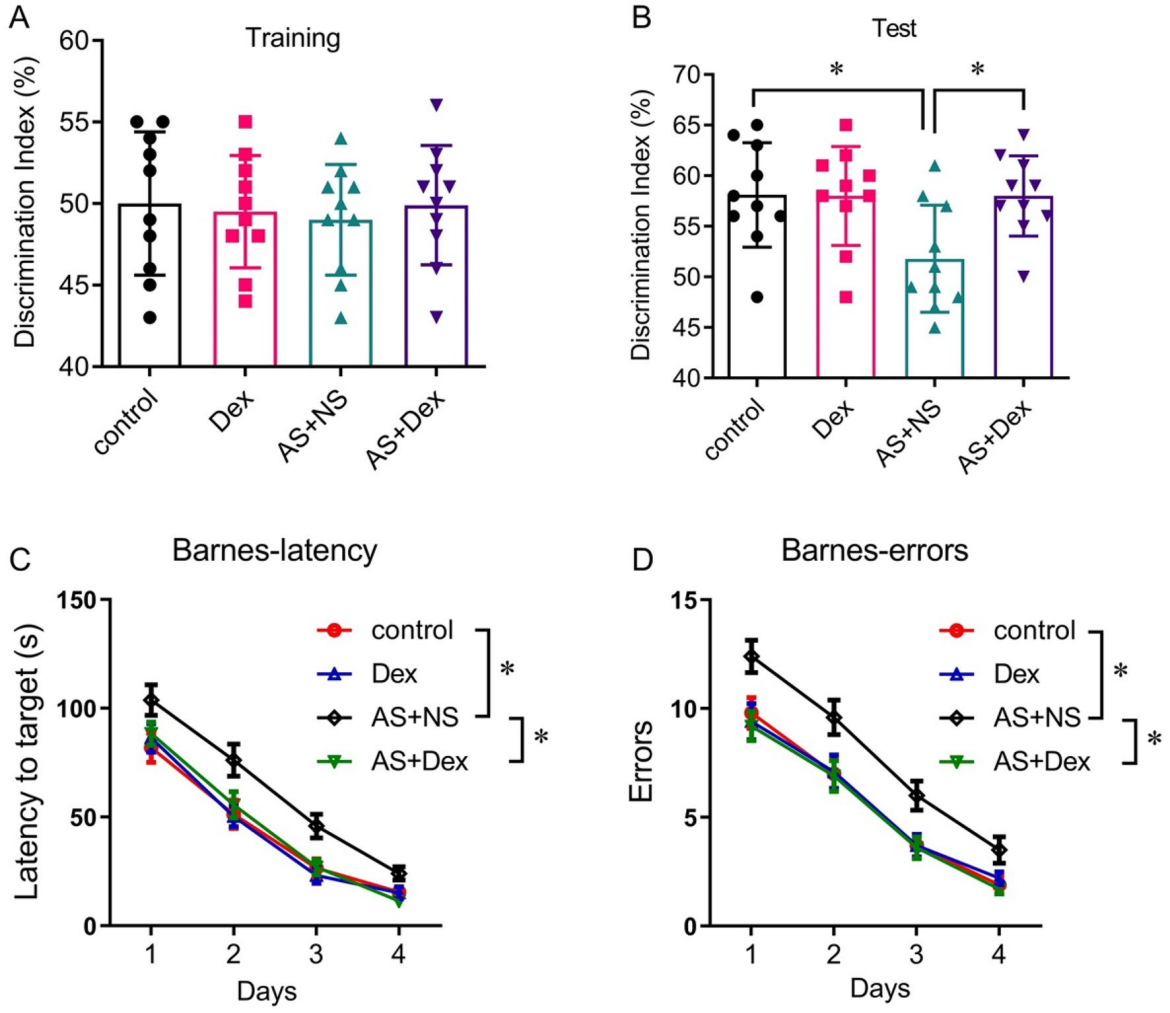
Dex attenuated learning and memory behavior of the acute stress mouse by NOR and Barnes maze test. **A**, **B** Discrimination index of each group of mice in training and test experiments in the NOR. **C**, **D** Average latencies and errors to the target of four group mice in the Barnes maze test (*n* = 10/group). Data are expressed as mean ± SEM. *p < 0.05

NOR test results showed that Dex intervention improved AS-induced cognition impairment (Fig. 3A, B). In the Barnes maze test, the latency and errors in the AS + NS group were significantly increased compared with the control group, which was reversed by Dex administration (Fig. 3C, D).

Taken together, these results suggested that Dex treatment improves anxiety behavior and learning memory impaired by AS in mice.

### Species abundance and diversity of intestinal gut

**Fig. 4:**
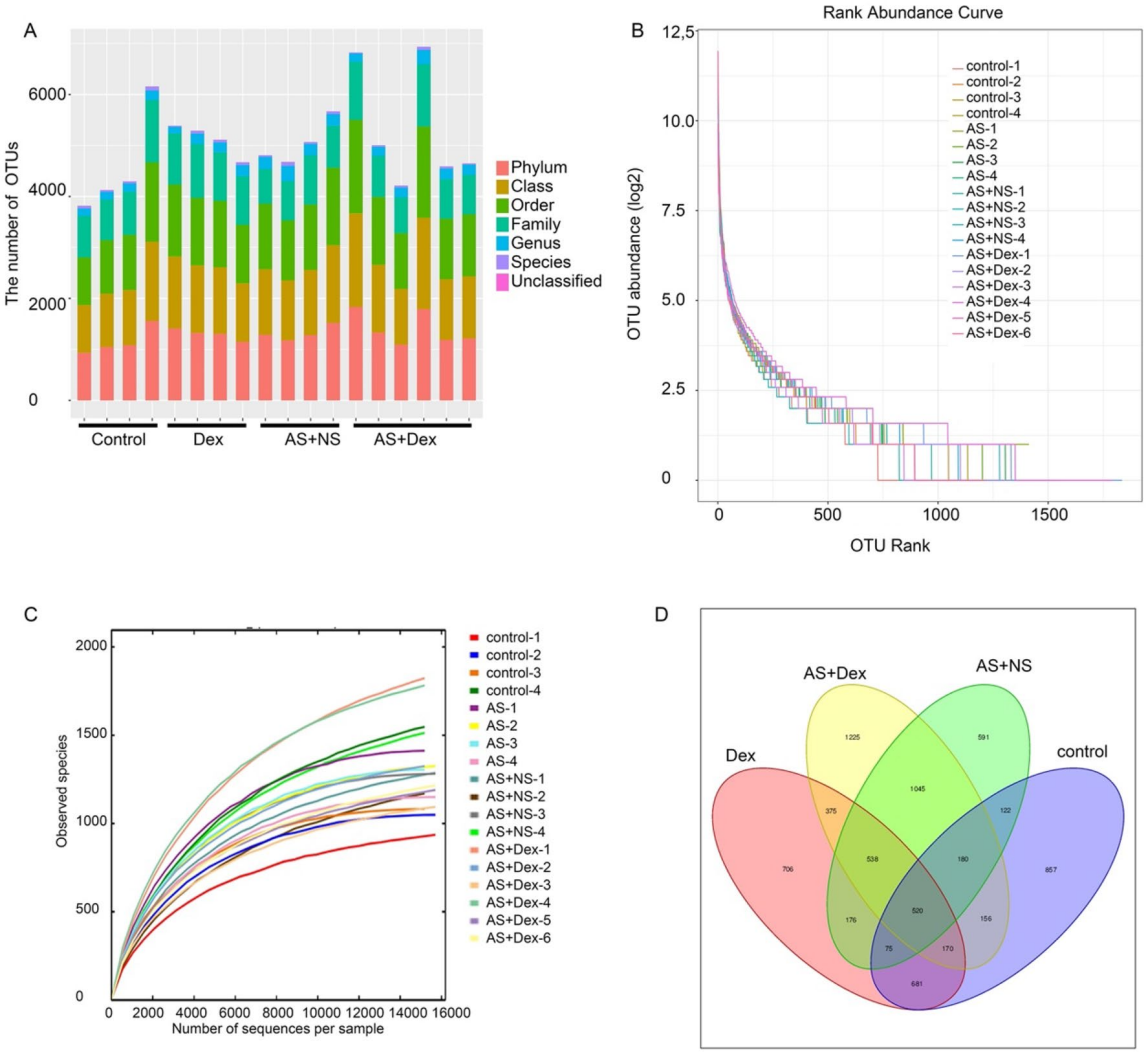
Species abundance and diversity of intestinal gut. **A** Community structures of different microbes at the Phylum, Class, Order, Family, Genus, Species, and Unclassified level in the four groups, different colors indicate distinct microbe (*n* = 4–5/group). **B** Rank abundance curves represented every individual sample (*n* = 4–5/group). **C** Dilution curves of all of the samples (*n* = 4–5/group). **D** Venn diagram depicting richness and overlap of OTU in four groups (*n* = 4–5/group)

A total of 913,000 raw readings were obtained from 18 samples, with an average reading of Phylum (23,542), Class (23,542), Order (23,542), Family (16,286), Genus (3611), Species (772) and Unclassified (5) (Fig. 4A). The Rank abundance curve indicated the distribution of species richness and evenness was stable (Fig. 4B). Rarefaction curve of each sample was also steady, indicating the sequencing data were sufficient to reflect the diversity of most species in our current samples (Fig. 4C).

A petal diagram was drawn with the four groups’ OTUs, which indicated the distribution and diversity of sequencing data in each group was consistent (Fig. 4D). Taken together, these results showed that the diversity of most of the microbiome contained in the samples was obtained.

**Fig. 5:**
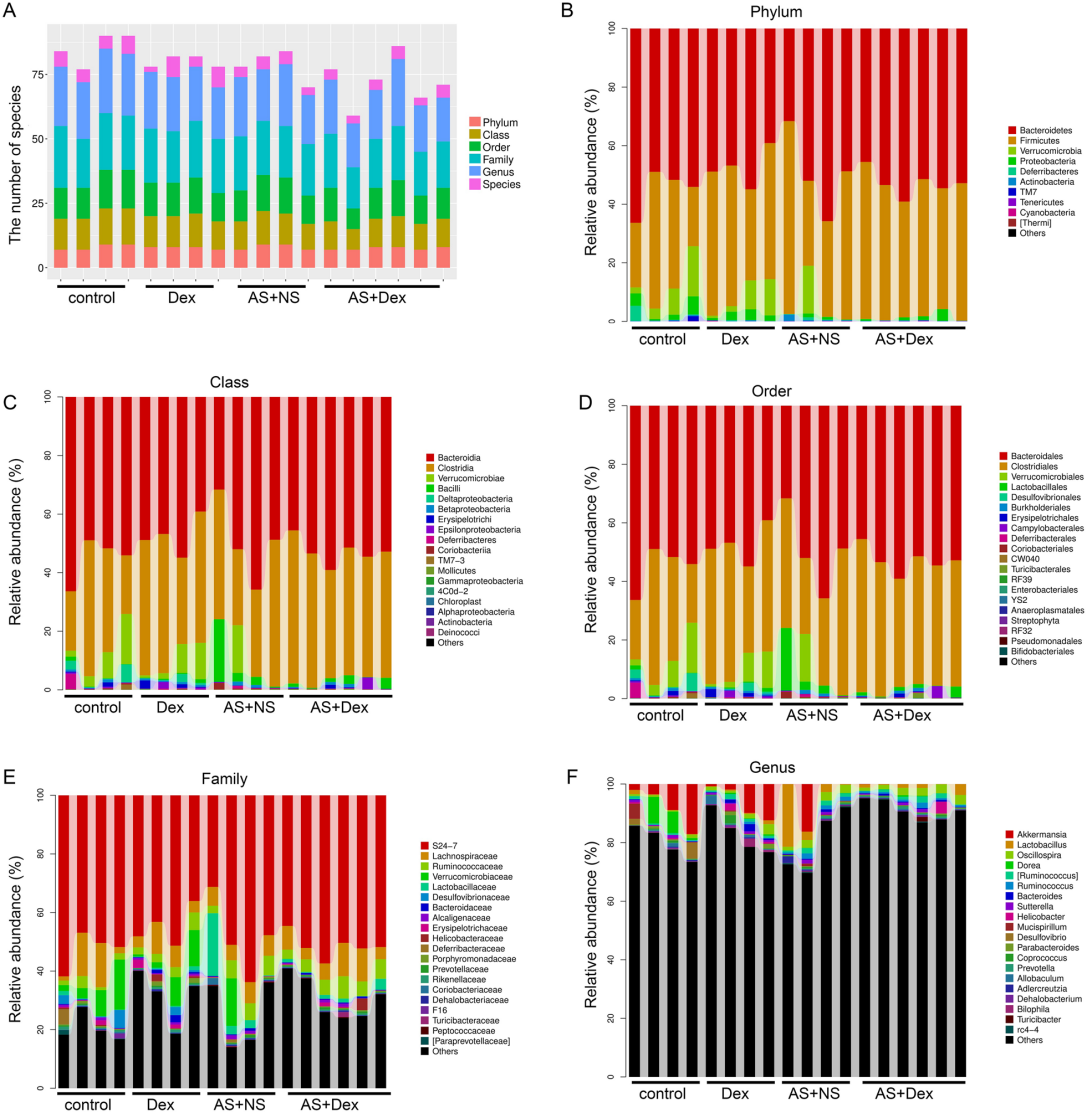
Comparison of relative taxa abundance among the control, Dex group, AS + NS group, and AS + Dex group at Phylum, Class, Order, Family, Genus, and Species levels. **A** Bar chart of relative taxa abundance among the three groups at Phylum, Class, Order, Family, Genus, and Species levels. **B** Bar chart of relative taxa abundance among the four groups at Phylum levels. **C** Bar chart of relative taxa abundance among the four groups at Class levels. **D** Bar chart of relative taxa abundance among the four groups at Order levels. **E** Bar chart of relative taxa abundance among the four groups at Family levels. **F** Bar chart of relative taxa abundance among the four groups at Genus levels

There was no significant difference in the distribution of the microbiomes contained in each of the four groups at the Firmicutes, Class, Order, Family, Genus, and species levels (Fig. 5A). Compared with the control group (31.53%), the percentage of Firmicutes in the Dex group was 43.68%, indicating Dex treatment could upregulate Firmicutes. However, Dex treatment could not upregulate Firmicutes after AS stimulation (44.53% vs. 45.7%) (Fig. 5B). At the Class level, the main proportions were the clostridia class and the verrucomicrobia class, but there was no significant difference between the four groups (Fig. 5C). Dex treatment did not alter the percentage of proteobacteria class under normal condition [control group (0.45%), Dex group (0.58%)], while Dex treatment significantly reduced AS-mediated upregulation in proteobacteria class (6.9% vs. 1.6%). In addition, the percentage of deferribacteres class was downregulated in the AS + NS group (0.03%) compared with the control group (0.28%), which was reversed by Dex treatment (0.03% in the AS + NS group vs 0.73% in the AS + Dex group) (Fig. 5C).

At the Order level, the main proportions were the Bacteroidales Order and the Clostridiales Order, but there was no significant difference between these four groups (Fig. 5D). Interestingly, the percentage of Lactobacillales Order in the AS + NS group (6.8%) was greatly increased compared with the control group (0.45%), while Dex treatment significantly reduced the percentage of Lactobacillales Order under AS condition (1.30% vs. 6.8%) (Fig. 5D).

At the Family level, the main proportions were the S24-7 family and Lachanospiraceae family, but there was no significant difference between the four groups (Fig. 5E). Dex treatment significantly reduced the percentage of Lactobacillaceae Family under AS condition (6.8% in AS + NS group vs. 1.30% in AS + Dex group) (Fig. 5E).

At the Genus level, the main proportions were the Unclassified family, but there was no significant difference between these four groups (Fig. 5F). Dex treatment significantly reduced the percentage of Lactobacillus under AS condition (6.8% in AS + NS group vs. 1.30% in AS + Dex group) (Fig. 5F).

### Differences in intestinal flora structure between groups

**Fig. 6:**
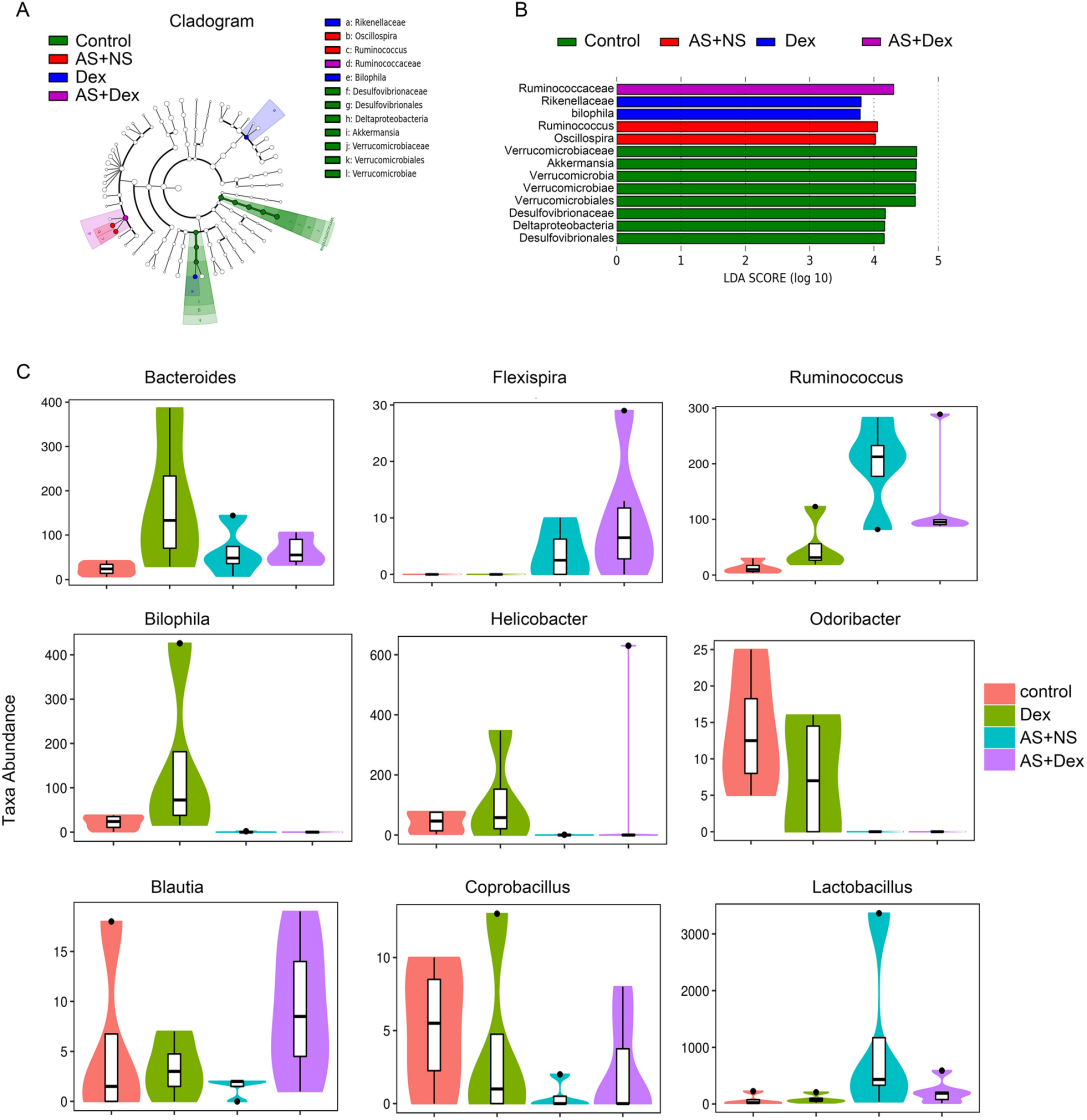
Enriched flora analysis in four groups. **A** Relative abundance of the flora is shown by each circle’s diameter. LDA scores greater than 3 when comparing all groups at the Genus level. **B** Linear discriminant analysis effect size (LEfSe) 10 analysis was set up with 0.05 as the alpha value for the Kruskal–Wallis and Wilcoxon test and then selected OTUs with LDA scores above 3 at the Genus level. **C**–**K** Metastat different significance analysis was used to analyze the Top1–9 species in four groups

According to the composition and sequence distribution of each sample at taxonomic rank, the LefSe and LDA analysis was performed for the abundance differences between the four groups. The results showed there were significant differences and enrichment in the group samples (Fig. 6A, B). Desulfovibrionaceae, Desulfovibrionales, Deltaproteobacteria, Akkermansia, Verrucomicrobia Verrucomicrobiae, and Verrucomicrobiales were enriched in the control group at the species level. Rikenellaceae and Bilophila species were enriched in the Dex group. Oscillospira and Ruminococcus species were enriched in the AS + NS group. Ruminococcaceae species were accumulated in the AS + Dex group (Fig. 6A, B).

Metastat different significance analysis was further used to analyze the Top1–9 species in the four groups (Fig. 6C–K). Compared with the control group, Bacteroides were increased after Dex treatment in control mice, but there were no differences in the AS + NS group and AS + Dex group mice (Fig. 6C). Compared with the control group, Flexispira and Ruminococcus were strongly upregulated in AS + NS group, while Dex treatment did not affect Flexispira and Ruminococcus (Fig. 6D, E). Dex treatment increased Bilophila and Helicobacter in normal mice, but could not reverse AS-mediated reduction in Bilophila and Helicobacter (Fig. 6F, G). Similarly, Dex treatment could not reverse AS-mediated reduction in Odoribacter (Fig. 6H). Interestingly, Dex treatment had no effects on *Blautia* and *Coprobacilus* under normal conditions. However, Dex treatment completely reversed the AS-mediated reduction of *Blautia* and *Coprobacilus* (Fig. 6I, J). Surprisingly, Lactobacillus was significantly increased in AS + NS group mice, which was reduced by Dex treatment (Fig. 6K).

Taken together, these results indicated that acute stress could induce intestinal microbiome disorders in mice, which could be partially improved by Dex intervention.

## Discussion

In this study, we analyzed the possible mechanism of Dex in improving stress-induced anxiety-like behavior and cognitive impairment from the perspective of the microbe–gut–brain axis. We found that intraperitoneal injection of Dex significantly improved acute restrictive stress-induced anxiety-like behavior, recognition, and memory impairment. By the analysis of intestinal flora, we found that acute stress caused intestinal flora disorder in mice. Dex intervention improved the flora disorder to a certain extent.

Dex is a potent selective α2-adrenergic receptor agonist that is used for sedative therapy. Recent studies have shown that Dex maintains the stability of the patient's perioperative hemodynamics and reduces the occurrence of respiratory inhibition [18]. In addition, Dex has anti-anxiety and brain neuroprotection effects [19, 20]. In this study, we found that Dex significantly improved anxiety-like behavior and cognitive impairment induced by acute stress. In a variety of animal models, Dex has been proven to inhibit inflammation and effectively reduce the release of inflammatory cytokines [21]. For example, Dex significantly reduced brain inflammation in mice induced by lipopolysaccharide, reduced cytokine levels, and improved abnormal symptoms in mice [22, 23]. Acute or chronic stress causes an inflammatory response to release pro-inflammatory factors such as TNF-α, which mediate the inflammatory response of the central nervous system, damage neurons, and promote their apoptosis or degenerative changes, ultimately causing a decline in learning and memory abilities [24–26]. Therefore, stress-induced inflammation is an important factor in the occurrence of anxiety-like behavior and cognitive impairment. However, the mechanism of how Dex reduces inflammation in the central nervous system is still unclear. The possible mechanisms may be as follows: Dex activates the cholinergic anti-inflammatory pathway to reduce the inflammation by releasing cholinergic transmitters and by activating the central α2 adrenergic receptor, which inhibits the release of pro-inflammatory factors, such as IL-1β, IL-6, and TNF-α [27, 28]; Dex regulates inflammatory response cells, including macrophages and neutrophils [29–31].

Gut microbes interact with the central nervous system through the microbe–gut–brain axis and affect brain functions and behaviors [32–35]. Obesity, autism, depression, and Parkinson’s disease are closely related to the number and composition of intestinal microbes [36–38]. We found that Dex intervention did not significantly change the intestinal flora of unstressed rats, but Dex intervention significantly reduced the proportion of Deltaproteobacteria in the intestinal flora of acute-stress mice. Deltaproteobacteria is a type of sulfate bacteria that is part of the normal gut microbiota. Increased levels of them may lead to the development of colitis, which may be related to the production of hydrogen sulfide [39]. For example, Imazalil exposure could induce inflammation of the colon, which was characterized by inflammatory cell infiltration, increased levels of lipocalin-2 in feces, and increased Deltaproteobacteria [40].

Oscillospira is a common but rarely cultivated genus of intestinal bacteria. Recent studies on the human gut microbiota have proved its potential for host health [41]. Acute stress mice mainly enriched in the Oscspira and Ruminococcus genus. Ruminococcaceae were enriched in Dex-treated acute stress mice. It was suggested that Dex mainly inhibited the expansion of Oscillospira. Keren et al. showed that in patients with gallstones, the average relative abundance of Oscillospira was higher [42]. Oscillospira was negatively correlated with the indicators of obesity or metabolic disorders in epidemiological studies [41], suggesting that the influence of Dex on Oscillospira is related to the regulation of metabolic balance.

We found that Dex intervention significantly reversed the acute stress-mediated decrease in *Blautia*. *Blautia* is a genus of anaerobic bacteria with probiotic properties, widely present in the feces and intestines of mammals. *Blautia* plays a role in metabolic diseases, inflammatory diseases, and biotransformation [43]. According to phenotypic and phylogenetic analysis, some species of Clostridium and Rumenococcus have been reclassified as *Blautia* [44]. *Blautia* plays an important role in maintaining the balance of the intestinal environment and preventing inflammation by up-regulating intestinal regulatory T cells and producing short-chain fatty acids [45]. *Blautia* has been shown to block NF-κB expression and IL-8 secretion, and induce colonization resistance to pathogens; *Blautia* can also metabolize curcumin to desmethyl curcumin, which has been found to have better neuroprotection and anti-inflammatory effects [46, 47]. These findings indicate that *Blautia* has a protective effect on inflammation-related diseases [48, 49].

We found that Dex induced a significant reduction in the genus of Coprobacillus mediated by acute stress. It has been reported that compared with obese subjects, Coprobacillus has a higher content in healthy subjects and is proposed as a new type of probiotic [50, 51]. Interestingly, the Lactobacillus genus, which is a probiotic, had a significant increase in expression after acute stress stimulation and was down-regulated to a normal level after Dex intervention. Although lactic acid bacteria are commonly used probiotics, it has been reported that the abundance of certain types of lactobacilli in obese humans has increased [52, 53], indicating that the effects of lactobacilli on metabolic and inflammatory diseases may be condition-specific[54].

## Conclusion

This study found that Dex improved acute stress-induced anxiety-like behaviors and cognitive impairment. Dex intervention changed the composition of the intestinal flora of acute stress mice, stabilized the ecology of the intestinal flora, and significantly increased the levels of *Blautia* and *Coprobacillus*. These findings suggest that Dex attenuates acute stress-impaired learning and memory in mice by maintaining the homeostasis of intestinal flora. However, a prospective cohort study is needed to further prove our findings.

## Data Availability

Not applicable.
